# The Moral of the Tale: Stories, Trust, and Public Engagement with Clinical Ethics via Radio and Theatre

**DOI:** 10.1007/s11673-016-9766-5

**Published:** 2017-01-06

**Authors:** Deborah Bowman

**Affiliations:** St George’s, University of London, London, UK

**Keywords:** Public engagement, Ethics and theatre, Clinical ethics, Ethics and the media, Narrative, Trust

## Abstract

Trust is frequently discussed with reference to the professional–patient relationship. However, trust is less explored in relation to the ways in which understanding of, and responses to, questions of ethics are discussed by both the “public” and “experts.” Public engagement activity in healthcare ethics may invoke “trust” in analysing a moral question or problem but less frequently conceives of trust as integral to “public engagement” itself. This paper explores the relationship between trust and the ways in which questions of healthcare ethics are identified and negotiated by both “experts” and the public. Drawing on two examples from the author’s “public engagement” work—a radio programme for the British Broadcasting Corporation and work with a playwright and theatre—the paper interrogates the ways in which “public engagement” is often characterized. The author argues that the common approach to public engagement in questions of ethics is unhelpfully constrained by a systemic disposition which continues to privilege the professional or expert voice at the expense of meaningful exchange and dialogue. By creating space for novel interactions between the “expert” and the “public,” authentic engagement is achieved that enables not only the participants to flourish but also contributes to trust itself.

## Introduction

Ethical concerns and questions frequently provide the framework within which scientific development and medical advances are discussed publicly. It is commonplace for those of us with “ethics” in our professional titles to be invited to consider the ethical implications of a scientific innovation or medical development. Sometimes, these invitations take the form of *ex post facto* comment on a “crisis,” such as pandemics (Garrett et al. 2009), failures of care or the patient safety agenda (Pronovost and Faden 2009), or on “hard cases” that have usually arisen where there is unresolvable conflict. Frequently, the discussion tends towards providing a moral lens for crystal-ball gazing in which prospective possibilities are interrogated and analysed, although there are (deontological) exceptions (Gregory 2003).

Public consideration of ethics in science and medicine is notable for three reasons. First, it is predominantly concerned with bioethics i.e. consideration of the moral dimensions of science and medicine as shaped primarily by research and innovations. It is, in many ways, a mirror that reflects the history of Western bioethics itself and the ways in which it emerged as a response to professional power in medicine and science (McCullough 2002). Clinical ethics i.e. the experiences and moral preoccupations of individual practitioners, clinical teams, patients, and families are less often explored as part of public engagement activities. Secondly, public engagement work tends to be expert-led notwithstanding its place in non-specialist settings. Broadcasting outlets, newspapers, festivals of ideas, and public lectures are commonly the preserve of those who have specific credentials and, by implication, who speak or write with authority. Finally, ethical discussion has a particular function in contributing to the ways in which trust in science, medicine, and its practitioners is conceptualized, represented, and understood.

This paper explores the relationship between trust and the ways in which questions of healthcare ethics are identified, described, presented, and negotiated, in public discourse. For the purposes of this paper, trust is considered to depend on the capacity of people to recognize in each other a commitment to understanding, sharing perspectives, and seeking meaning. It is trust that is embedded in attention and care rather than attaching to a specific issue or question. Trust is conceptualized as existing where diverse perspectives and multiple meanings are acknowledged, explored, and sought. Drawing on two examples from the author’s own practice of so-called “public engagement” work—a popular radio programme for the British Broadcasting Corporation (BBC) and work with a playwright and a professional London theatre—the paper interrogates and challenges the ways in which “public engagement” is characterized. It is argued that public engagement in questions of ethics is constrained by a systemic disposition which continues to privilege the professional or expert voice at the expense of meaningful exchange and dialogue; an approach that can be considered an example of epistemic injustice (Carel and Kidd 2014). It further suggests that form matters and that trust is integral, but overlooked, as a conceptual basis for public engagement activities. The novel approach in each project, it is argued, not only allows for new ways of sharing, talking, and thinking which are both predicated on and engender trust but also offers a distinctive and richer concept of “public engagement” which seeks to challenge the epistemic privilege of the professional voice or perspective. Such an approach has much to offer those who are charged with, or interested in, engagement about questions of ethics in healthcare, medicine, and the biosciences.

## Ethics and Public Engagement

The form and function of public engagement have not been much considered, although recently a small number of papers have focused on the discourse of “public bioethics” (Miah 2005; Moore 2010). Where ethical consideration, debate, and discussion occur, consequentialist perspectives tend to dominate, experts proliferate, and, as a result, the boundaries within which ethics is understood are delineated. As a function of the form, content, and function of the ways in which the subject of bioethics and its significance are presented and represented, a partial picture emerges and opportunities for rich exchange and open dialogue are missed.

In what ways are the form, content, and function of public engagement in ethics limiting? The form too often defaults to that of the “talking head” or “sage on the stage.” Sometimes, expertise is carefully curated or reverts to those with a remit to comment on, and consider, matters of medical and bioethics. Such an approach may lead to performance of a quasi (or even, an actual) regulatory function, as much as it facilitates public engagement. The growth and inclusion of ethics as a subject for exploration at literary, science, and ideas festivals tends conservatively towards the lecture model or, sometimes, the interview or facilitated conversation in which an expert or panel of experts share perspectives or debate a question or an issue. Despite recent interest in social media as a more inclusive tool for public engagement (Regenberg 2010), the commonest approach remains, in the author’s experience, a tightly managed format that privileges academic and professional credentials, intellectual confidence, and articulacy (Stebbing 2009). Frequently, the physical distance at a venue between the speaker(s) and audience reinforces the metaphorical gap. Carefully controlled engagement between those who speak with those who listen (who may well have personal knowledge or experience of the issue being discussed) limits and forecloses dialogue.

The content of public engagement events and activities in ethics is, of course, varied and wide-ranging. However, it tends towards being issue-led and it is often focused on the prospective and technological developments (Pickersgill 2011). It is, perhaps inevitably, concerned with “the big questions” and the new, innovative, or cutting edge. A familiar media trope is the ethicist invited to discuss the implications of some novel technology, treatment, or biomedical research. Another common source of material is hard cases, many of which are characterized by conflict and may have reached an impasse, perhaps ending up in the courts for judicial determination. There may be elision between the legal, political, and ethical analyses and participants are often encouraged to argue for a specific perspective or adopt a “position” (Bowman 2015). Clinical ethics, stories of the everyday and the routine feature rarely. Participants who are uncertain or ambivalent are not often invited. Attention to process and the seeking of common ground or shared values may yield to displays of individual intellectual gymnastics and powerfully expressed arguments.

Finally, the function of public engagement activities warrants closer examination. Questions about the unintended or unconsidered impact of scientific development and medical advance are a familiar starting place. Ethics, even well done, is offered as a containing device which is frequently concerned with the consequences and impact of an innovation, choice, or decision. The ethical discussion provides an opportunity to check the normative content of something new and/or difficult (Wynne 2006; Moore 2010). It may make refer explicitly to the need to balance risks and benefits and, in so doing, begs questions and makes assumptions about the value and motives of a scientific development or medical decision. There is reassurance to be sought and provided in the discussion. Diverse perspectives will be acknowledged and, to some extent, considered. The act of the debate or discussion itself is a represented as a “good thing” and an “important job, done well.” It is the approach of a civilized and sophisticated society that embraces progress, is mindful of the perils of unchecked professional power, and open to plurality and complexity. A perception that has been further bolstered by the attention and incentives attached to public engagement activities.

What might be possible if the form, content, and function of public engagement activities in ethics were revisited? This paper describes two specific projects that are distinct from the approach that predominates, namely the creation of a long-running radio series for BBC Radio 4 and the production of a new play for a major London theatre. Each project is described and discussed with particular reference to the ways in which their form, content, and function enabled a new type of public engagement. The approach of these two projects, it is argued, creates space for a relationship of trust in which notions of expertise are challenged, new perspectives are celebrated, and collaborations beyond credentials thrive.

## De-familiarizing “Hard Cases” and Building Trust: “*Inside the Ethics Committee*” and “*Test Case*”

In 2005, I received a call from a producer at the BBC. She had an idea for a programme that she wanted to discuss. She was hoping, she explained, to create a new series that would share the stories of cases that had been considered by clinical ethics committees in the United Kingdom. Her intention was to use real cases and to ensure that the stories would be told by the people who were involved: patients, clinicians, family members, other professionals, and, sometimes, the members of the ethics committee that had originally considered the case. The stories would be presented, unedited, to a panel in the studio which would explore the themes and questions that arose from the case and model discussion and analysis of an ethico-clinical problem.

I was, I confess, hesitant, even discouraging, in that initial conversation with the producer. My response was characterized by concern and caution. I expressed reservations and asked large numbers of questions about anonymity, consent, the ways in which relationships would be nurtured and participants protected. The producer patiently and thoughtfully addressed each one in turn. Would I be interested, she asked, in working with the production team on the programme? My role would be as a consultant to the series and as a regular panelist on individual episodes. Impressed by her consideration, intelligence, and commitment, I asked for more written information and time to think about it. A number of further conversations took place and I met members of the production team in person. Eventually, albeit with some apprehension, I began working on the first episode of *Inside the Ethics Committee* for BBC Radio 4.

**Fig. 1:**
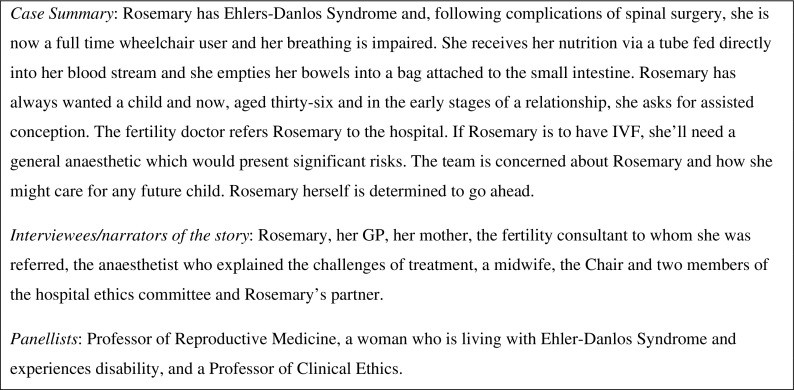
Example of an episode of *Inside the Ethics Committee*, Series 9, Episode 1

There have since been twelve series of *Inside the Ethics Committee* and being part of the programme has been a transformative experience that has, for me, challenged notions of expertise in ethics and taught me a great deal. Whatever the subject of the episode, the format is consistent. Stories are told in three parts and by those who were involved. Different perspectives are offered on common events with equal time being given to the patient’s account as to those of the professionals involved. Some stories originate with the team involved and some from the patient and/or family members. In some cases, the professionals and the patient approached the production team together. The story unfolds progressively with three pauses in the narrative in which panel members discuss what they have heard. The panel members are invited because of the diverse perspectives they bring and have regularly included a non-professional member. The programme has covered many different subjects and experiences, ranging from mental capacity to transplants and from fertility to end of life care. Figure 1 below describes the outline of an episode from series nine of the programme (British Broadcasting Corporation 2013).

The series has been a success for the BBC, attracting large numbers of listeners and high approval ratings from audiences and critics (Mahoney 2011). The programme has won awards and, most gratifyingly of all, it has prompted many people to write to the production team and the participants in response to what they have heard. The production team is as concerned with process as with output. The programme follows a three act narrative structure whereby the story unfolds and prompts “real-time” immediate responses from the panelists in the studio. The form, content, and function of the programme are distinct in a number of ways.

First, the patient’s account comes first and is shared in an unmediated way as he or she chooses. The experience and perspective of the patient form the crux of what is explored. The stories that are shared are unmediated; first person accounts are its essence. On only one occasion has a story been voiced by an actor; a decision taken in order to protect other family members. Even in that case, the words the actor spoke were the unedited words of the person involved. Instead of “cases” or “histories,” people share the stories of their lives. This is not a semantic distinction. For example, in the programme summary set out in Figure 1 above, the producer was approached by Rosemary herself; an articulate woman who had previously competed in Paralympic sport, she was committed to sharing her story and challenging assumptions regarding disability and parenthood. The broadcast edition of the programme begins with Rosemary’s voice explaining who she is, how she is perceived by others, and why she wants a child. This is not a “reproductive ethics case” or a neat vignette that has been summarized by a professional. Instead, we hear Rosemary herself conveying the pain she has overcome, the emotional tug of longing for a child coupled with the fear of failing and the judgement of others. She is a vivid and complex presence from the outset. The perspective of professionals comes later and is contextualized within what we are coming to understand of Rosemary.

Secondly, the naming of the ethical question(s) or issue(s) is active and not “owned” or “pre-determined” by the form and/or claims to particular expertise. So, in the episode described in Figure 1 above, the audience and panel hear the clinical team’s perspective on Rosemary’s wishes after they have heard her speak for herself. The audience and panel members can gauge whether the professionals have understood what they know, from Rosemary herself, to be her priorities. They can assess whether and how the ethical questions are being adequately captured by the clinicians. It is not uncommon for the discussion to extend well beyond the boundaries that appear to have been defined by the clinicians in the story. What’s more, the clinical or expert perspective is often changing, uncertain, and varied. Different members of the same team or clinical service will share their own views and, as the story unfolds and new information becomes available. In the case of Rosemary, listeners learn of the differing views of the reproductive medicine team, including the specialist, a doctor who has known Rosemary for a long time, and the counsellor. The audience hears what gives them pause, prompts them to act, and how they think, with hindsight, about the decisions that they make. There is an openness that both acknowledges the complexity and uncertainty of clinical work and allows for the panel and audience to share in the search for meaning(s) and understanding whatever their personal perspective and experience.

Thirdly, there is the composition of, and discussion by, the panel. Equal weight is afforded to the professional and the “lay” voice. In clinical ethics, the involvement of patients and lay participation is a challenge and success is mixed (Reiter-Theil 1998, 2003; Fournier et. al. 2009; Neitzke 2009). Inside the Ethics Committee has achieved not only participation but it has sought, from the outset, to put the experience of patients and the accounts of those who have been directly involved at the centre.

As the summary in Figure 1 describes, the panel is deliberately constructed for diversity. The programme has undergone a transition in the ways in which it draws on expertise for the discussions. The panels have, since the outset, been multidisciplinary drawing from a wide range of specialists, including, medics, ethicists, nurses, social workers, lawyers, chaplains, and charity workers. However, from series four, the programme has included non-professional experts: patients and family members who have experienced the condition or situation discussed in the programme. As I have written elsewhere (Bowman 2015), the addition of this perspective to the programme has immeasurably enriched the discussion. It redraws the boundaries of what types of knowledge matter in ethical discussions. Theoretical analyses are accompanied by personal insights and technical objectivity sits alongside subjective experience to create a richer and balanced exploration.

All panel members are equal in contributing to the discussion and the discussion is evolving, allowing people to demonstrate how their perspective is changing or changed as a result of new information or a point raised by another panelist. There is a dynamism and interactionism to the programme which allows for, perhaps even demands, nuance and ongoing consideration. It is, in the author’s experience, an approach to “public engagement” that is unique, relational, and inherently ethical. It recognizes that trust is its currency: for the participants, the discussants, the audience, and the discipline (Eastwood 2010).

It is striking what a difference is made by the programme’s approach and choices. It is not uncommon for the clinicians involved to hear, via the production team or the recordings themselves, new information or to be offered a perspective that has, to date, not been part of their discussions and decision-making. The format makes this possible. The stories are recorded with great care and consideration. The recordings are intimate and personal encounters: usually, the producer will travel to an individual’s home or other location of his or her choice. The sometimes inhibiting influence of busy hospitals, overbooked clinics, disorienting wards, and professional power is absent. The patient and his or her family members are free to speak as they wish in a familiar and safe environment. It is a physical and metaphorical space in which stories can breathe and be heard afresh. There is time to share complex narratives without the imperative to sift those accounts into “histories” or “case studies.” It is a pure and rich narrative.

The professionals are also shown the same care and attention as they share their stories and perspectives. This is vital as there is often disagreement within the clinical team and capturing that divergence honestly and respectfully is fundamental to the authenticity of the programme. All those involved are invited and encouraged to participate if they wish: the production team works hard to ensure that hierarchy and professional affiliations do not preclude capturing the range of perspectives on a situation or problem. Whether it is sharing the stories of patients, family members, or professionals, trust is essential both to the creation and form of the programme. That the team manages to build trust in such sensitive situations and are given permission to share often painful stories where conflict and loss are recurrent themes is a reflection of their commitment to an inclusive, respectful, and authentic approach where process and content both matter equally.

The approach to ethical analysis and discussion in the programme is not wedded to one model or theoretical perspective. It is grounded in the unmediated narrative. The attention to the individual story, both in its content and in the ways in which it is shared, leads to a rich discussion that extends beyond the conventional academic analyses. There may be a single word, a silence, or an exchange that changes meaning and informs responses. The relational is the essence of the discussions and the complexity of the specific is explored in detail. Priority is afforded to finding ways of working together in the face of uncertainty or conflict. Therapeutic, personal, and professional relationships, and time, attention, and care to process all matter.

Of course, the creation and broadcast of a programme is but the beginning and the response to *Inside the Ethics Committee* has been enriching. The most valued response has come from individual listeners who have been moved and stimulated by the programme. From the beginning, the production team and I received letters, emails, and even phone calls from people who wanted to continue the conversations begun during the programme. It is not only listeners who are changed by the programme. Those who participate are too. In the episode offered as an illustrative example for this paper, we received news that a fellow panelist who is lives with Ehlers-Danos syndrome had decided to have a child of her own following her participation in the programme. She described the way in which the experience of hearing and reflecting on Rosemary’s story and interrogating her own responses shaped her subsequent personal choice.

The producer and I have spoken about the series at many events where we are often touched and surprised by the programme’s impact. I have learned from every single communication that I have received and each has seemed to be a more effective act of “public engagement” than other events that were so badged. It has created sufficient trust for people to share their own stories, to reflect on and develop their own views, and to offer their perspectives on the widest range of ethical questions. It has been a transformative privilege and a highlight of my academic career.

## Is This a Dilemma I See Before Me? On Working with Theatre

The theatre offers a creative and engaging way to reach wide and diverse audiences. Drama is inherently concerned with different perspectives and multiple interpretations. Theatre arises from, and presents, conflicts of all kinds, making it well-suited to the exploration of ethical questions (Nisker et al. 2006; Fearnow 2007; Lewando Hundt et al. 2011; Freeman 2012). Theatres exist in communities and will often have a programme of engagement and educational activities to accompany productions. Theatrical work and drama provide a unique space for a creative approach to public engagement in, and exploration of, ethical questions that, I suggest, both depends on and develops trust as hierarchies are eliminated, perspectives shared and reflexivity fostered.

Last year, The Donmar Theatre in Covent Garden, London, approached me to discuss a new play that was in development and for which they had received some funding from the Wellcome Trust. The play was *Elegy* by Nick Payne (2016). I was sent an early draft of the script; a beautifully-written, tightly-crafted meditation on the future of medicine, the nature of identity, and the meaning of care. It is a three-hander in which a couple and a doctor navigate the possibilities of treatment for an unnamed degenerative condition. The treatment is novel and life-saving, but it has one significant side-effect: the loss of memory for the period between diagnosis and treatment. Choosing treatment also means losing all recollection of, and love for, a spouse. It is a play that asks the ultimate ethical question: what if? It does so with skill, subtlety, and sophistication. I had no hesitation in agreeing to work with the production team.

*Elegy* is a non-linear drama that explores questions of identity, the ethics of innovative medical treatments, and trust in relationships (both therapeutic and personal). It is an intimate drama set at an unspecified time in the future and it received its world premier at the Donmar Theatre in April 2016. In working on the play and being part of a programme of activities to support the production, I was reminded that creating performance is an active process which yields rich engagement whereby notions of “default” expertise are constantly challenged, allowing discussion of fundamental ethical questions to flourish in ways that not only enhance our understanding of the specific issue at hand but also serve to strengthen trust and to create a form of engagement that is rich and enduring.

**Fig. 2:**
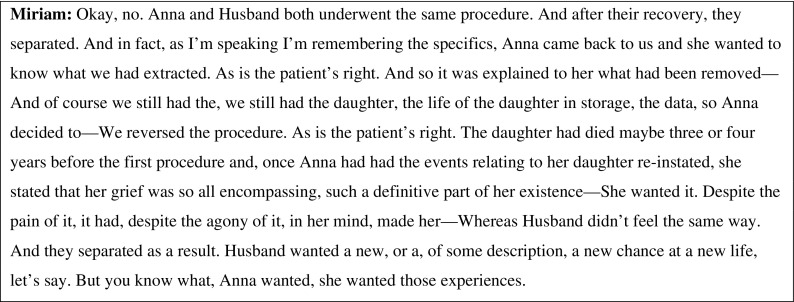
Excerpt from *Elegy* (Payne 2016)

My involvement in the project lasted approximately six months. It included commenting on the script, taking part in rehearsals, creating role play exercises for the characters, writing the programme essay, joining the education team in its work, and participating in a number of events. At opening night, I watched the audience as closely as I watched the production. In barely seventy minutes, the play covered complex ethical terrain absorbingly and effectively. There was no “expert” exposition or specialist debate. The honed narrative did the work: emotions and intellect were engaged, philosophical questions were contextualized in moving personal stories, and the sometimes remote nature of academic bioethics discourse became immediate and urgent. The brief excerpt in Figure 2 below offers a glimpse of the ambitious moral questions that characterize *Elegy*. The character speaking is Miriam, a doctor who is able to offer the treatment that removes disease but also memory.

Alongside the collaboration with the creative team, the work with young people was a particularly powerful experience that was predicated on the principles of co-creation (Walmsley 2013). The *Take the Stage* programme run by The Donmar Theatre offers those studying at local colleges and schools a unique opportunity to engage with both the content and form of theatre. Many of the participants had never been in a professional theatre before and some of them had never visited the West End despite living all their lives in London. Led by professional actors and theatre practitioners, groups of young people spend a two month period on a range of activities, exercises, and workshops exploring the ideas and form of the production. Equal attention was afforded to the production’s content as to the creative approach. Each group drew on these experiences to devise a short response piece to the play. Those response pieces were performed on the main stage at The Donmar as the culmination of the *Take the Stage* programme.

My experience of working with the education team on *Take the Stage* was outstanding and unforgettable. None of the sixty or so young participants had any formal experience of ethics. Many of them described themselves as “not academic” or even “rubbish at school work.” Some of them confessed that they “hated science.” Yet, each and everyone of them offered energy, enthusiasm, interest, and unique perspectives on the ethical questions raised in the play and explored during the workshops. The vocabulary was not theoretical or academic, but the concepts on which the debates and discussions were predicated were familiar: these young people talked freely about “rules” and their relationship to them, they were quick to challenge assumptions about consequences but recognized that weighing risks and benefits was inherent to moral analysis, they referred to being “a good person” and offered their own suggestions for the virtues that mattered to them, the relational aspects of ethical choices and decision-making was a recurrent theme, especially when thinking about how decisions are informed by, and also affect, family members and friends. Within moments, they grasped the differences between the brain, the mind, and identity, and why those differences might matter. They shared their own experiences of ethical problems in their lives and described vividly the ways in which professionals and society can respond to, and sometimes misunderstand, a teenager. They were natural story tellers and shifted between the literal and metaphorical in explaining and representing their perspectives. They invoked both the real and the imagined as effective ways of considering moral questions.

In between workshops, I occasionally received tweets, emails, and messages asking me specific questions or testing out some of the participants’ ideas. The programme culminated in the performance, on the Donmar stage, of the response pieces these young people had developed to the play and during their work. Watching the growth in confidence, the development of ideas and the talent on stage was immensely moving. In keeping with the principles of *Take the Stage*, the pieces were sophisticated in both theatrical form and content. It was public engagement at its most meaningful. These young people, who only eight weeks ago had never heard of bioethics or clinical ethics, offered work that was creative, sophisticated, and original. The play itself was the spring board for the exploration of big questions and complex ideas. The *Take the Stage* programme had created the space, and offered support to produce a response that was grounded in their own reactions to the production and its themes. It was a genuine dialogue and the process, although facilitated by professionals and experts, was led by the young people themselves. At every point, they were in charge and the brief was to walk alongside them as they decided the directions in which they wanted to go. It was an approach that was as far from the expert-led public engagement events (that none of these participants would likely attend anyway) as it could be. And, it was all the better for being so distinct and for challenging notions of “public engagement.”

## Trust and Public Engagement: Concluding Thoughts

Trust has been frequently discussed with reference to the therapeutic relationship and in relation to the wider “professional project” (Larson 1977) of medicine, often in conjunction with considerations of power, privilege, and regulation (Clark 2002; O’Neill 2002; Hall 2005; Allsop 2006; Evetts 2006; Pfadenhauer 2006). However, trust is less explored in relation to the ways in which understanding of, and responses to, questions of healthcare ethics are described and discussed by both the “public” and “experts”; although trust has been considered in relation to particular modes of communication, such as social media (Snyder 2011). Within professional relationships, trust, or its absence, creates a particular context for the negotiation of norms and the determination of what is considered to be an “ethical question.” Public engagement work in relation to healthcare ethics may cite trust as a consideration in the analysis of a moral question or ethical problem but less frequently extends to conceiving of trust as integral to the interactions and activities that are categorized as “public engagement” and the potential implications for narratives of trust in relation to moral questions, health, illness, and society.

The two examples of working with radio and theatre described in this paper offer, I suggest, a different approach to developing a trusting relationship which, in turn, facilitates a novel way of understanding public engagement. Each project—working on *Inside the Ethics Committee* and contributing to the production of *Elegy*—is concerned with the creation and exploration of narrative. That is not incidental to fostering trust nor is it an accidental choice of form. It is an approach that is inherently committed to attending to the experiences and perspectives of people irrespective of status, expertise, training, or position. It is necessarily non-hierarchical and inclusive, seeking and celebrating diversity, plurality, and complexity. Both *Inside the Ethics Committee* and *Elegy*, although different in their focus, approach, and medium, are committed to capturing the nuanced, uncertain, and demanding nature of moral questions as they are experienced and interpreted by the widest range of individuals, be they real people or characters in a play.

Trust is at the centre of each project. For the programme and the play to have any impact or make a connection, they each have to engage an audience. Trust depends on a pact between the creators of the work and the audience members that is entirely voluntary: no one has to listen to the radio or attend the theatre. Neither can exist in any meaningful way without the other: it is a symbiotic and evolving relationship with trust at its centre. The audience is offered ideas but the interpretative work is theirs. What resonates and what engages will change not only between performances or broadcasts but also between people who watch or listen to each iteration. And that, it is suggested, is the essence of effective public engagement: the confidence to trust the audience to take the questions and ideas in the programme or production and do with them what they will.

What’s more, in the projects described in this paper, trust is understood to be dependent on fostering an ongoing relationship with the audience and public at large: it is not secured in a one-off or one-way transaction. Trust is nurtured and dialogue is encouraged. Opportunities for discussion and the deliberate facilitation of sharing without privileging expert perspectives or professional power are integral to the approaches discussed *ante*. Although the phrase is an academic one that has never been used in relation to these projects, they are fundamentally concerned with recognizing and addressing the different types of epistemic injustice (Carel and Kidd 2014, 2016) that is evident not only in healthcare settings but even in well-intentioned public engagement work in ethics. Epistemic injustice may, argue Carel and Kidd (2014, 2016) manifest in two ways, namely testimonial injustice and hermeneutic injustice. In the former, the attention and regard afforded to the accounts of non-specialists, particularly patients, are compromised or deficient, often because of the assumed impact of illness. Hermeneutical injustice refers to a collective failure in appreciation or understanding and is commonplace in healthcare because it may be difficult to convey or make sense of experiences of illness. Both Inside the Ethics Committee and Elegy challenged epistemic injustice. By offering rich first person perspectives that flourish beyond the confines of the clinic and its norms, the testimony about, and meanings of, illness and disability are brought into focus. The form, whether of radio programme or drama, allows for that focus and flourishing, acknowledging the privileged position of the professional and redressing the balance deliberately and creatively.

Interrogating the predominant discourse of, and approach to, public engagement in the ethical dimensions of healthcare is essential if we are to develop public awareness of, and responses to, moral questions. It is also integral to whether the field of ethics in healthcare thrives (Simonson 2012). Unless and until we attend to questions of expertise, structural constraints on effective public engagement, and the significance of nurturing public trust in the field itself, there is a risk that participation will be limited, understanding thwarted, and ultimately meaningful engagement compromised irrespective of the numbers of claims or aspirations to foster “public engagement.” It is a challenge for us all and it is one we cannot afford to ignore for reasons that are more essential and important than any external imperatives or incentives to demonstrate “impact” in academic scholarship.
